# Ageing vision and falls: a review

**DOI:** 10.1186/s40101-018-0170-1

**Published:** 2018-04-23

**Authors:** Liana Nafisa Saftari, Oh-Sang Kwon

**Affiliations:** Department of Human Factors Engineering, Ulsan National Institute of Science and Technology, Ulsan, 44919 South Korea

**Keywords:** Falls, Ageing, Vision, Visual motion perception, Postural balance, Vection

## Abstract

**Background:**

Falls are the leading cause of accidental injury and death among older adults. One of three adults over the age of 65 years falls annually. As the size of elderly population increases, falls become a major concern for public health and there is a pressing need to understand the causes of falls thoroughly.

**Main body of the abstract:**

While it is well documented that visual functions such as visual acuity, contrast sensitivity, and stereo acuity are correlated with fall risks, little attention has been paid to the relationship between falls and the ability of the visual system to perceive motion in the environment. The omission of visual motion perception in the literature is a critical gap because it is an essential function in maintaining balance. In the present article, we first review existing studies regarding visual risk factors for falls and the effect of ageing vision on falls. We then present a group of phenomena such as vection and sensory reweighting that provide information on how visual motion signals are used to maintain balance.

**Conclusion:**

We suggest that the current list of visual risk factors for falls should be elaborated by taking into account the relationship between visual motion perception and balance control.

## Background

Falls are the second leading cause of accidental deaths after road traffic accidents worldwide. It is estimated that 646,000 people die from falls worldwide [1]. According to the WHO global report published in 2007 [1], 28–35% of people aged > 65 years old fall each year and this prevalence increases to 32–42% for people > 70 years old.

The increase in fall risk in the elderly is unavoidable because physical, sensory, and cognitive functions deteriorate with age. Numerous studies have reported various factors related to fall risks [2, 3] in an attempt to clarify the causes of falls and to propose methods of fall prevention [3, 4]. The ability of balance and gait control [5], musculoskeletal functions [6, 7], cardiovascular functions [8–10], vestibular functions [11, 12], somatosensory functions [13, 14] and visual functions [4, 15, 16] have been suggested to be the major factors responsible for the increase in fall risks in older adults.

As demonstrated in the classical study by Lee and Aronson [17], in which young stationary observers fell in response to the visual motion of a whole scene, visual information plays a critical role in balance control. Given that visual functions deteriorate with age, it is not surprising that many studies have found correlations between the ageing of visual functions and falls in elderly people [4, 15, 16]. However, the majority of the existing studies focused on the correlations between fall risks and performance in simple visual detection or discrimination tasks such as visual acuity, contrast sensitivity, and stereo-acuity [18–23]. Consequently, the list of visual risk factors for falls in the literature often does not include the ability of visual motion perception as a significant contributor to fall risk [20, 24–27]. It is a critical omission, because it has been well documented since the seminal study by Lee and Aronson [17] that balance control is strongly influenced by visual motion signals [17].

We will first review the characteristic changes in vision as a result of ageing, and we will review the relationship between visual functions and fall risk in older adults. We will then discuss the mechanism by which visual motion signals are directly used in balance control by reviewing phenomena such as vection and sensory reweighting. Finally, we will introduce three relevant phenomena that could elaborate our understanding of how visual functions are related to fall risk.

## Visual functions and falls

Visual function unavoidably deteriorate with age, and the deterioration of visual function in turn increases fall risk [15, 20, 28, 29]. In this section, we will first provide an overview of anatomical and functional changes in the visual system in the elderly and will then review the studies that examined whether and how specific deficits in visual functions are correlated with fall risks. Both the effects of ageing on visual functions and the relationship between the visual functions and fall risk are summarized in Table 1. Notably, although numerous studies have reported a significant correlation between diverse visual declines and falls, the correlation between the ability of processing visual motion signal and fall risk has not been reported. It is an important gap in the literature because visual motion signals induce an automatic reaction of the balance control system. We will discuss the relationship between visual motion perception and balance control further in the next section.

**Table 1:** The effects of ageing on visual functions and the relation between visual functions and fall risks

Visual function	Author	Ageing effect	Relation to fall
Visual acuity	Lord and Dayhew [52]	Visual acuity declined as age increased (*P* < 0.01, *n* = 156)	Having visual acuity larger or equal to 0.6 logMAR was shown to have 1.83 higher risk of multiple falls (95% confidence interval (CI) = 0.98–3.39, *n* = 156).
	Coleman et al. [28]	Visual acuity change in 5.6 years from baseline study was: loss of 1–5 letters = 31.9%, loss of 6–10 letters = 20.9%, loss of 11–15 letters = 7.7%, loss of > 15 letters = 4.6%, *P* < 0.0001, *n* = 4275	Loss of acuity in reading letters in Bailey-Lovie chart was shown to have 1.85–2.51 higher risk of multiple falls (*P* < 0.05, *n* = 4275).
	Willis et al. [22]	Subjects with worse visual acuity and visual impairment relatively older than subjects with normal vision (mean age 58.0, *P* = 0.07 and 74.2, *P* < 0.01 respectively)	Increase 1 unit scale in logMAR was shown to have 1.6 higher rates of failure in standing on foam surface with eyes closed (95% confidence interval (CI) = 1.12–2.42, *n* = 4393).
	Ivers et al. [20]		Having visual acuity worse or equal to 20/30 was shown to have 1.2 higher rates of multiple falls (95% confidence interval (CI) = 1.1–1.3, *n* = 3299).
	Koski et al. [112]		Having poor distant visual acuity was shown to have 2.3 higher risk to major injurious fall (*n* = 979).
	Klein et al. [113]		Having poor binocular acuity was shown to have 2.02 higher risk to multiple falls (95% confidence interval (CI) = 1.13–3.63, *n* = 3722).
	Ivers et al. [114]		Having corrected visual acuity worse than 20/60 was shown to have hazard ratio of 8.4 for risk of hip fractures (95% confidence interval (CI) = 1.5–48.5, population attributable risk = 27%).
	Dolinis et al. [50]		Having worsen vision in past 5 years was shown to have 1.34 higher risk of multiple fall (95% confidence interval (CI) = 1.06–1.68, *n* = 1285).
	Felson et al. [115]		Having poor vision was shown to have 2.17 relative risk of hip fractures (95% confidence interval (CI) = 1.24–3.80, *n* = 2633).
	Dargent-Molina et al. [116]		Having visual acuity worse or equal to 2/10 was shown to have 2.0 higher risk to hip fractures compared to participant who have visual acuity worse than 7/10 (95% confidence interval (CI) = 1.1–3.7).
	Cummings et al. [18]		Poor visual acuity was not associated to risk of hip fracture.
	Grisso et al. [117]		Loss of distant vision such as fail to recognize someone’s face across the room was shown to have 4.8 higher risk to hip fractures (95% confidence interval (CI) = 1.4–16.2) *N* = 174.
	Ivers et al. [60]		Having visual acuity worse than < 20/100 was shown to have 2.4 times higher risk to hip fractures (95% confidence interval (CI) = 1.0–6.1).
	Friedman et al. [48]	Study population with median age 72.6 (range = 65.9–86.3) have median visual acuity − 0.02 logMAR (range = − 0.19– 1.7)	Population’s visual acuity was not significant in predicting falls, *N* = 2211.
	Bongue et al. [47]		Distance visual acuity in fallers and non-fallers did not show significant relation (OR 0.97, 95% confidence interval (CI) = 0.92–1.02) *N* = 1759.
	Kulmala et al. [51]		Having visual acuity worse than 1.0 was shown to have 1.5 higher risk of falls (95% confidence interval (CI) = 0.6–4.2) *N* = 428.
	Tromp et al. [53]		Loss of distant vision such as fail to recognize someone’s face across the room was shown to have 2.6 times higher risk to multiple falls (95% confidence interval (CI) = 1.8–3.8) *N* = 1285.
	Lamoureux et al. [21]		Presenting visual acuity worse than 20/40 but better than 20/200 in the better eye (0.3 < logMAR < 1.0) was not significantly associated with falls. *N* = 3261
Contrast sensitivity	Ivers et al. [20]		A 1-unit decrease at 6 cycle per degree in contrast sensitivity was shown to have 1.3 times higher risk of multiple falls (95% CI 1.1–1.6), *n* = 3299
	Szabo et al. [118]		The mean fall risk index score in the AMD cohort (3.20) was significantly greater than that of the non-AMD cohort (3.20 and 1.21, respectively; *P* < 0.001).
	de Boer et al. [19]		Having lower integrated contrast sensitivity was shown to have 1.53 times higher risk of multiple falls (95% CI 1.03–2.29)
	Tiedemann et al. [56]	Subjects’ mean of edge contrast sensitivity (MET) score 18.8, *n* = 688, mean age = 80.1, SD = 4.4	Contrast sensitivity (mean = 18.8 dB) correlates with 6-m walking speed (mean = 1.07 m/s) *r* = 0.29, *P* < 0.001, *n* = 688.
	Delbaere et al. [57]	Study population’s mean Physiological Profile Assessment Risk (PPA) fall risk score is 0.8 (*z* score) mean age = 76.9 SD = 5.1	Having higher score in PPA falls risk score was associated with slower walking speed (*P* = 0.029, *N* = 44).
	Wood et al. [23]	Subjects’ mean of edge contrast sensitivity (MET) score 16.6, *n* = 76, mean age = 77, SD = 6.9	Reduced contrast sensitivity was significantly associated with increased rates of falls, injurious falls and other injurious events such as collision with an object. (*P* < 0.001, *P* < 0.014, *P* < 0.037 respectively)
	Lord and Dayhew [52]	Contrast sensitivity declined with increase of age (*r* = − 0.37, *P* < 0.01, *n* = 156)	Having poor visual contrast sensitivity below or equal to 18 dB was 1.76 times more likely to fall (95% confidence interval (CI) = 0.94–3.27), *n* = 156.
	Cummings et al. [18]		Having decrease per 1 SD unit in contrast sensitivity was 1.2 times more likely to have hip fractures (95% confidence interval (CI) = 1–1.5), *N* = 9516.
	Lord and Fitzpatrick [58]		Contrast sensitivity in fallers (mean = 17.5 dB) was significantly worse than non-fallers (mean = 18.7 dB) (*P* < 0.001).
	Lord and Menz [119]	Subjects’ mean of edge contrast sensitivity (MET) score 20.3 dB, *n* = 156, mean age = 76, SD = 5.1	Contrast sensitivity was significantly correlated with postural sway on foam (*r* = − 0.36, *P* < 0.01 *n* = 156).
Depth perception	Lord and Dayhew [52]	Depth perception declined with increase of age (*r* = − 0.32 *P* < 0.01, *n* = 156)	Having poor depth perception was 2.51 times more likely to have multiple falls (95% confidence interval (CI) = 1.40–4.51, *n* = 156).
	Lord and Menz [119]	Subjects’ mean of howard dohlman score 2.7 cm, *n* = 156, mean age = 76, SD = 5.1	Depth perception was significantly correlated with postural sway on foam (*r* = 0.30, *P* < 0.01, *n* = 156).
	Cummings et al. [18]		Participant in lowest quartile of distance depth perception was 1.5 times more likely to have hip fractures (95% confidence interval (CI) = 1.1–2.0), *N* = 9516.
	Felson et al. [115]		Moderately impaired vision in one eye and good vision in the other was 1.94 times more likely to have fracture.
	Friedman et al. [48]	Study population with median age 72.6 (range = 65.9–86.3) have median stereoacuity 1.8 arcsec (range = 1.4–2.96)	Population’s stereoacuity was not significant in predicting falls, *N* = 2211.
	Lamoureux et al. [21]		Having bilateral visual acuity such as poor visual acuity in one eye and mild or moderate visual acuity in the other eye was 2.1 times more likely to have multiple falls (95% CI 1.4–3.1). *N* = 145
	Lord et al. [46]	Presbyopia makes elderly depends to bifocal glasses and multifocal glasses wearers have poor performance in depth perception when viewing the rods through the lower segments of their glasses (*P* < 0.001, *n* = 87)	Wearing multifocal glasses was 2.29 times more likely to have multiple falls (95% confidence interval (CI) = 1.06–4.9).
	Ivers et al. [60]		Having no depth perception was 6.0 times more likely to have hip fractures 95% CI 3.2, 11.1).
Visual field	Broman et al. [15]		Missed visual field per 10 points increased number of bumps as much as 17% (*P* < 0.0001).
	Freeman et al. [63]		Missed central visual field per 5 points increased risks of fall 1.06 times while missed peripheral visual field per 4 points increased risk of fall 1.08 times. Worse visual field scores were associated with the risk of falling (95% CI 1.03–1.13).
	Ivers et al. [20]		Missed visual field per 5 points was 1.8 times more likely to falls.
	Coleman et al. [16]		Severe binocular visual field loss was 1.50 more likely to falls (95% CI: 1.11–2.02).
	Friedman et al. [48]	Study population with median age 72.6 (range = 65.9–86.3) have median visual field loss 17 points (range = − 0.19–1.7)	Population study visual field was not significant to predict fall risk (*n* = 2211).
	Ivers et al. [114]		Having visual field loss was 5.5 times higher the risk of hip fractures.
	Ramrattan et al. [64]		Bilateral visual field loss was associated with fall accidents (*P* < 0.05 *n* = 109).
	Owsley and McGwin [120]	Population’s useful field of view composite score mean was 32.8 (SD = 12.6)	Lower scores on visual attention/processing speed were significantly related to poorer scores on the performance mobility assessment (*P* = 0.04), *N* = 342.
	Turano et al. [121]		Loss in the overall visual field per 10 point missed was associated with 1.22 times increase in the number of bumps (*P* < 0.0001). Visual field loss was not associated with the number of orientation errors. *N* = 1504
	Patino et al. [66]		Loss in central visual field was 2.36 times more likely to higher the risk of falls. Loss in peripheral visual field was 1.42 times more likely to higher the risk of fall. (95% CI 1.02–5.45 and 1.06–1.91 respectively). *N* = 3203
	Black et al. [65]		More extensive field loss in the inferior region was 1.57 times more likely to higher risk of falls and 1.80 times more likely to falls with injury (95% CI, 1.06 to 2.32 and 1.12 to 2.98 respectively).
	Glynn et al. [122]		In patients attending glaucoma clinic, visual field impairment glaucoma patients of 40% or greater was associated with fall risk (OR, 3.0; 95% CI, 0.94 to 9.8).
	Lord and Webster [123] 1990		Fallers have more dependency to visual field when asked to align vertical rod to the true vertical (*P* < 0.02, *N* = 136).

### Ageing vision

The decline in many visual functions in older adults can be directly attributed to anatomical changes of the eyeball. The anatomical changes reduce the quality of sensory inputs to higher-level visual processing and, as a result, visual functions deteriorate. However, some functional declines cannot be fully explained by anatomical changes. Declines of computational efficiency and compensatory heuristics in the higher-level visual processes are responsible for those functional changes. In this section, we will review anatomical changes of eyeball first and functional declines of vision in general.

#### Anatomical changes

Ageing is accompanied by structural changes to the eyeball. The weight and cross-sectional area of the lens in the eye change throughout the lifespan. As we age, the lens that is responsible for the change of focal distance of the eyes, allowing it to focus on objects at various distances by changing its shape, becomes heavier and thicker and loses its elasticity. The resistance of the lens to external force exponentially increases with age resulting in presbyopia, a condition characterized by a decreased ability to focus on near objects [30]. Changes due to ageing also can be observed in the ciliary muscle, a smooth muscle surrounding the lens that changes the shape of the lens during accommodation for viewing objects at various distances. The diameter of the ciliary muscle in the relaxed accommodation condition negatively correlates with age [31].

It has been well documented that the aberrations of the eye increase with age, which causes the deterioration of spatial vision [32]. To determine the main factor of the age-related increase in the aberrations, Artal et al. [33] measured the aberrations in both the corneal surface and the complete eye in subjects across a range of age groups. The results indicated that aberrations of the cornea increased with age, although the size of the increase was too small to explain the total aberrations of the eye. The main difference between young and older subjects was the coupling between corneal and internal aberrations. In the eyes of younger subjects, the corneal aberrations were compensated by the internal aberrations, which made the total aberration smaller than the corneal aberrations. In the eyes of older subjects, the total aberrations were larger than corneal aberrations, indicating no sign of a compensation mechanism.

Anatomical changes of the eyeball due to ageing cause a reduction in the quality of visual inputs to the central nervous system and contribute to the decrease of visual function performance. For example, ocular aberrations in older eyes result in a decrease in modular transfer function (MTF), which transfers object resolution and contrast to a retinal image. This poor quality retinal image contributes to the decrease in contrast sensitivity [34]. However, anatomical deterioration may not completely explain the decrease in contrast sensitivity, as neural mechanisms have also been reported to contribute to the decrease in contrast sensitivity with age [35].

#### Functional changes

The majority of visual functions decline with age. In the 1990s, a large-scale project, termed the Salisbury Eye Evaluation project, was conducted to examine visual ageing [36]. In the study [36], the visual performance of 2520 older adults aged 65 to 84 years old was examined in various visual tasks. Visual acuity, contrast sensitivity, glare sensitivity, and visual field size were found to decline linearly with age.

Haegerstrom-Portnoy et al. [37] measured spatial vision, high- and low-contrast acuity, contrast sensitivity, disability glare, glare recovery, color vision, stereopsis, and visual fields to understand the effect of ageing on visual functions. The results from 900 subjects aged 58 and 102 years old indicated that the high-contrast acuity was relatively well maintained until the age of 65 to 70 years old, and began to decline above the age of 70. Meanwhile, all the other visual functions declined with age. Notably, the authors found that a single exponential function well fits the data representing the changes in spatial vision across the range of ages. Betts et al. [38] measured contrast sensitivity under a range of external noise levels. The contrast threshold in older adults was significantly higher than in younger adults when the external noise level was low; however, it was comparable when the external noise level was high. This result suggests that lower contrast sensitivity observed in older subjects was mainly due to the higher level of the internal noise rather than the deteriorated ability to filter out external noise. Sloane et al. [35] measured contrast sensitivities as a function of luminance levels across four spatial frequencies. In general, the contrast sensitivity was lower in older adults than in young adults. However, more importantly, the size of the differences was significantly larger in the low luminance levels compared to the high luminance levels, which suggests that the optical mechanisms cannot fully account for the decline in the contrast sensitivity in older adults. In an additional experiment, the effect of temporal flickering on contrast sensitivity with age was measured by presenting a target grating that flickered at 7.5 Hz. The results revealed that flickering affects contrast sensitivity differently across age groups, which again suggests that the age-related decline in spatial vision was partially due to the changes in neural processing rather than purely optical.

Bian and Andersen [39] examined how ageing affects one’s judgment of egocentric distance. Egocentric distance is the perceived distance between an observer and a location in the 3D world. The results indicated that younger observers had a tendency to underestimate the egocentric distance, while older observers did not. One possible explanation for this result is that older observers have accumulated more knowledge regarding egocentric distance in real-world scenes and consciously or unconsciously use this knowledge to correct the estimation bias. The role of past experience in egocentric distance judgment was also examined in a study that compared egocentric distance judgments between athletes and non-athletes [40].

Motion perception is another visual function that is affected by ageing. Snowden and Kavanagh [41] studied how the ability to motion perception changes with age. The low-speed threshold was higher for older adults compared to young adults across wide spatial frequency levels (0.5 to 4 cycles/degree), and the speed discrimination threshold (i.e. the minimum speed differences of two stimuli to be distinguished) at a range of speeds (0.1°/s to 10°/s) was also higher for older adults. The coherence threshold (i.e. the minimum percentage of coherently moving dots to reliably detect motion directions), for the random dot kinematograms stimulus, was higher for older adults when the stimulus speed was relatively low (< 2°/s). However, the coherence thresholds for high-speed random dot kinematograms were almost identical for older and young adults. Their results suggested that deficits in motion perception could not be fully accounted for by contrast sensitivity or visual acuity. Betts et al. [42] found that for high-contrast large-size motion stimuli, older adults exhibited higher sensitivity than young adults. This counter-intuitive finding could reflect the centre-surround antagonism of neurons in middle temporal area [43]. Motion signals from the peripheral receptive field effectively suppressed the signals from the central receptive field in young adults, whereas the suppression effect was diminished in older adults, resulting in higher sensitivity for large high-contrast stimuli. As listed above, the effect of ageing on the ability of motion perception varies widely across the different stimuli and tasks. This inconsistency demonstrates the complex mechanisms involved in motion perception.

### Correlation between the decline in visual functions and fall risk

The decline in the visual functions of older adults impairs gait and balance control, and consequently increases fall risk [4, 15, 16]. A large-scale survey study, termed the Blue Mountain Eye Study, examined the relationship between visual functions and fall risk in 3299 older adults [20]. The study found that visual acuity, contrast sensitivity, glare sensitivity, and visual field size were significantly correlated with fall risk. Eye diseases such as a cataracts and glaucoma were associated with falls. The study also confirmed that age, sex, psychotropic drug use, and history of stroke are associated with the number of falls. In this section, we will discuss in detail the effect of a specific visual function on fall risk.

#### Visual acuity and fall risk

Reduced visual acuity is the most common visual impairment across age, gender, and ethnicity [44, 45]. As described in the previous section, the flexibility of the lens enables the eye to focus on near and far object, and the lens loses its flexibility and becomes harder with age. This causes many older adults to suffer from presbyopia (the inability to focus on near objects). And thus requires them to wear bifocal lenses. The bottom section of the bifocal lens allows wearer to focus in near distance while the upper section allows them to focus in a far distance. Wearing bifocal lenses may impair other visual functions, such as depth perception and contrast sensitivity, and cause falls [46].

Although some studies did not identify a significant relationship between visual acuity and fall risk [21, 47–49], the majority of studies have reported that older adults with low visual acuity have a higher risk of falls [28, 50–53, 124]. The reason behind these inconsistent outcomes is unclear. However, it may be worth noting that different visual acuity tests were used in different studies. In the studies that report a significant relationship between visual acuity and fall risks, the Bailey-Lovie chart [28], Landolt ring chart [51], Snellen chart [50, 124], low/high contrast letter chart [52], and face recognition test [53] were used. In the studies that did not report a significant relationship between visual acuity and fall risk, the Parinaud chart [47], Monoyer chart [47], LogMAR chart [21, 49], and the Early Treatment of Diabetic Retinopathy Study (ETDRS) scale [48] were used.

Visual acuity is also related to the vestibular contribution in controlling postural balance. Willis et al. [22] analysed the data from a large-scale survey of 4590 adults aged 40 years old and above and reported that the percentage of failure in maintaining balance was higher in individuals with uncorrected refractive errors even when visual and proprioceptive sensory inputs were not available (i.e. only vestibular signals were available). The authors suggested that the deteriorated visual inputs might weaken the efficacy of the vestibulo-ocular reflex, which is related to the efficacy of postural balance control by vestibular inputs.

#### Contrast sensitivity and fall risk

Contrast sensitivity in visual functions is the ability to discriminate between two luminance levels in a static image. Low contrast sensitivity, even with relatively high visual acuity, makes detecting hazardous objects in the environment more difficult, particularly at night [54]. Many studies have reported correlations between contrast sensitivity and fall risks. Contrast sensitivity function and the Melbourne Edge Test [55] are often used to measure contrast sensitivity. Contrast sensitivity function represents contrast thresholds for sine wave gratings across a range of spatial frequencies. Contrast sensitivity function not only related to the past occurrences of falls [20] but also predicted future occurrence of falls [19]. In studies focusing on gait and balance in the elderly, the most common contrast sensitivity measure is the Melbourne Edge Test [23, 56, 57]. In the Melbourne Edge Test, participants report the orientation of a line defined by the contrast between two abutted surfaces. Then, minimum contrast for edge detection is measured. Several studies using the Melbourne Edge Test found that low contrast sensitivity is associated with the occurrence of falls in the past [58] and the follow-up period of the test [52]. Furthermore, the contrast sensitivity correlates with performances in physical tasks, which is related to fall risk, such as the stand-to-sit task [14] and the choice stepping reaction time task [58].

#### Depth perception/stereoacuity and fall risk

Depth perception allows us to accurately construct spatial relationships between objects and ourselves, which helps us to navigate our movement in the environment [52, 59]. Depth information can be acquired by various cues that are often classified as monocular or binocular depth cues. Monocular depth cues, which include motion parallax, accommodation, blurring, and perspective cues, can be observed with one eye, whereas binocular depth cues, which include stereopsis and convergence, require both eyes. Stereoacuity tests are designed to measure the minimum difference between the images in the left and right eyes that can induce depth perception. The Howard-Dolman test and the Frisby Stereo test are commonly used to measure stereoacuity.

Lord and Dayhew [52] reported that performance in the Howard-Dolman test and the Frisby Stereo test was more strongly correlated with the occurrence of multiple falls in older adults than visual acuity, contrast sensitivity, and the size of the visual field. Poor stereoacuity was also associated with an increase in hip fractures [60], which is a common injury caused by falls [61]. Stereoacuity requires good quality of visual images from both eyes. Mono-vision condition in which one eye wears a contact lens for distance vision and the other eye wears a contact lens for near vision reduce stereoacuity and walking speed compared to both eyes having full distance vision correction [62]. And in another study, higher fall rates were found in subjects with high visual acuity in one eye and moderate or low visual acuity in the other eye [52]. The presence of a common factor in these two studies suggests that stereoacuity is related to fall risk.

#### Visual field and fall risks

Several studies have reported that the size of the visual field is strongly correlated with fall risk [15, 16, 20, 63, 64]. After re-examining the Salisbury Eye Evaluation data, Freeman et al. [63] found that visual field impairment was associated with self-reported fall occurrence. In another study, severe binocular visual field loss was associated with frequent falls during a 1-year follow-up period [16].

Different regions of the visual field have different effects on postural sway. A higher rate of falls was reported to be associated with the inferior region of the visual field, but not the superior region [65]; this result underlines the fact that, when dealing with the real-world environment, we depend more on the inferior region of our visual field. The risk of falls was associated with both central and peripheral visual fields [66]. Moderate to severe impairment in central vision was found to increase the risk of falls by 2.4-fold. While peripheral vision loss was found to increase the risk of falls by 1.4-fold. Straube et al. [67] also found that postural sway was less with central visual inputs than peripheral visual inputs when the sizes of visual field were the same. However, when they adjusted the size of the visual field in a way that the area of primary visual cortex representing the visual fields similar, there was no difference between central and peripheral vision in postural control. Based on this result, Straube et al. suggest that the contribution of visual stimuli to postural control is determined by the number of neurons in the primary visual cortex that are stimulated by the size of the visual field.

## Visual motion perception and balancing

Postural balance control is strongly affected by visual motion signals, because visual motion signals provide direct information regarding head movements [17, 68]. Considering this strong relation between visual motion perception and postural balance control, one may expect that deterioration in visual motion perception can cause a major deficit in balance control and increase the risk of falls. However, to the best of our knowledge, there have been no studies examining the correlation between visual motion perception ability and the risk of falls.

In this section, we will review studies investigating the mechanism by which visual motion signals generate reactive body movements. These studies can be classified into two topics: (1) studies related to vection and (2) studies related to sensory re-weighing, both of which will be discussed in detail.

### Vection and postural control

Vection is a sensation of illusory self-motion that occurs when an observer is exposed to a visual motion signal. It clearly demonstrates that perception of visual motion signals is directly related to postural balance control. Vection has been reported to occur in various directions: anterior-posterior direction/depth motion [69], left-right direction/circular motion [70], and clockwise-counterclockwise direction/roll motion [71].

The magnitude of vection can be manipulated by several factors. Brandt et al. [70] tested the effect of visual field size on vection using a circular vection stimulus that is produced by placing the subject inside a rotating drum with black and white vertical gratings. When the motion stimulus covers the entire visual field or the peripheral visual field without central vision, subjects reported the sensation of self-motion with a perceived speed that matched the speed of stimulus motion. However, when the motion stimulus only covered the central 30° without peripheral vision, subjects reported no sensation of self-motion. The results suggested that the motion signal in peripheral vision is critical to the perception of circular vection. The effect of the visual field size was also found in roll vection. Allison et al. [71] examined sensation of self-rotating motion (i.e. roll vection) in a tumbling room, in which subject sat in a stationary chair observing a furnished room rotating about the body roll axis. The rotation speed of the tumbling room and the degree of the visual field affected the magnitude of roll vection. When full-field view was allowed, the majority of the subjects reported a complete 360° body rotation. The percentage of subjects experiencing 360° roll vection decreased as the degree of visual field decreased.

Predictably, presenting stationary objects along with vection stimuli can diminish the magnitude of vection. A more important finding was that the effect of stationary objects on vection depends on the relative distance of between stationary objects and moving stimulus. Stationary objects presented closer to subjects than moving stimuli do not affect the magnitude of vection, whereas stationary objects presented further away than moving stimuli markedly reduced the magnitude of vection [72, 125]. The inhibitory effect of stationary objects was consistently found in forward/backwards vection [73]. This result is practical, because closer stationary objects can be perceived to move together with subjects, such as the interior of a moving car.

Vection and postural control are highly correlated as can be demonstrated by the fact that optic flow that generates vection [69] can also induce postural sway of an observer [74, 75]. In addition, the magnitude of vection is correlated with the size of the postural sway in various directions [76–78]. In depth vection direction, the magnitude of vection is positively correlated with postural sway. Postural body sway was measured while optic flow from random dot patterns was presented to participants [76]. Similarly, postural sway was also larger when the magnitude of the perceived roll vection direction was higher [78]. Recently, it was reported that dependence on vision in postural control predicts the strength of vection [79]. In detail, the dependence on vision in controlling upright posture was measured by subjects’ postural sway ratio in opened and closed eyes conditions. The magnitude of perceived vection was assessed by subjective rating given by subjects to vection stimuli that were introduced immediately after the postural control test.

The vection and postural sway correlation can be stimulated by direct visual motion and also by illusory visual motion caused by the motion after effect (MAE) [80]. The postural sway of participants was measured while being stimulated by the MAE. MAE stimulation began with adaptation to left or right motion of a random pixel array. This was followed by a black screen and test pattern which consisted of three conditions: a static random pixel array (expected to produce longer MAE), a dynamic random pixel array in which each pixel was randomly assigned to bright or dark every 16.7 ms (expected to produce shorter MAE), and a blank screen (expected to produce no MAE). MAE was perceived to be significantly longer in the static condition compared to the dynamic condition. Furthermore, postural sway was found to be larger in the static condition than in the dynamic condition.

With age, perceived vection declines but postural sway rate increases. In a study conducted by Haibach et al., postural sway was recorded in two age groups, young adults and older adults, with stimulation in the form of a virtual reality moving room. They were also asked to rate the perceived vection. The results revealed that older adults experienced smaller vection than young adults, but exhibited larger postural sway. The lower rate of perceived vection in older adults suggests that a reduction in proprioceptive feedback due to ageing may contribute to a larger postural sway [81].

Considering that visual motion stimulation is important in vection and postural control, in-depth investigation of visual cue components in this visual motion stimulation will be fruitful in aiding our understanding of fall risk with ageing vision.

### Sensory reweighting

In order to effectively estimate and control the state of body posture, the balance control system should integrate sensory inputs from proprioceptive, vestibular, and visual systems [82, 83]. There has been an interesting line of research suggesting that the degree by which the balance control system relies on each sensory input is not hard-wired, but adaptively changes. The phenomenon, which is termed sensory reweighting, suggests that the balance control system will rely on one sensory signal over the other as the sensory environment changes.

Sensory reweighting is observed in various contexts. Ageing alters the relative accuracy of sensory signals, and, consequently, older adults rely more on vision to control postural balance than on vestibular or proprioceptive signals [81]. Loss of vestibular function affects the way patients respond to the visual signals to control posture. Peterka [83] measured the size of postural sway as a function of the magnitude of visual signals. As the amplitude of the visual signal increased, normal subjects’ decreased gain to visual inputs was demonstrative of adaptive sensory reweighting. However, patients with vestibular loss exhibited a constant gain regardless of the magnitude of visual inputs.

Sensory reweighting can be classified as inter- or intra-modality reweighting. Inter-modality reweighting indicates a change in the gain of a sensory modality due to the magnitude change of motion signal of the same sensory modality [82, 84, 85]. Intra-modality reweighting refers to a change in the gain of a sensory modality due to the magnitude change of motion signal from a different sensory modality [84, 85].

An example of intra-modality reweighting was observed in an experiment conducted by Polastri et al. [85]. When an observer was surrounded by visual motion stimulation of constant frequency and standing on a platform that increased the sway amplitude, postural sway decreased relative to the platform. Another example of intra-modality reweighting is demonstrated when a randomly fluctuating visual motion stimulus was presented for 60 s and was introduced before presenting the main visual motion stimulus to observers [82]. The magnitude of sensory sway in response to the main visual motion stimulus was reduced. This result indicates that the postural control system re-adjusts the reliance on sensory modalities in response to the previous experience in an identical context.

Inter-modality reweighting was also demonstrated in the same study conducted by Polastri et al. [85]. When postural sway relative to the platform was decreased by an increase in the platform amplitude, postural sway relative to the visual stimulus increased as a result. Another example can be demonstrated by varied visual motion stimulation and constant touch motion stimulation amplitudes being introduced to observers simultaneously. Increased visual motion stimulation amplitude did not significantly affect the gain from sensory touch. However, when the condition was reversed (i.e. the amplitude of touch motion stimuli was varied and visual motion stimuli amplitude was set to constant), the increased amplitude of touch motion stimuli significantly affected vision sensory gain [84].

Sensory reweighting is slower in older adults when balance is being maintained. Hay et al. [86] investigated the mechanism by which older and young adults maintain their postural balance when the availability of visual and proprioceptive information was manipulated. When the proprioceptive inputs were perturbed by means of tendon vibration, both elderly and young adults exhibited a marked decrease of stability. A clear distinction between older and young adults was found when the perturbed proprioceptive inputs were reinserted. Young adults were able to rapidly integrate available information to control balance, whereas older adults were unable to utilize the reinserted sensory signals. These findings suggest that elderly adults are slower in adjusting weights on sensory modalities in response to their availability. A recent study also supports this result as, unlike young adults, older adults lose the ability to select stimulation such as galvanic vestibular stimulation (GVS) in order to reduce postural instability when perturbation of ankle and vision was introduced [87]. In a recent neuroimaging study on balance control, activity in the brains of older adults was distributed across several locations when somatosensory and visual information was absent, while in young adults the temporal-parietal region was more active, suggesting that in older adults sensory re-weighting demands more attention [88].

It is known that sensory reweighting is important in balance control and that ageing affects this ability even in healthy adults [89]. However, despite of older adults’ responses are generally poorer than young adults’ in a condition where sensory conflicts were presented, 1-h exposure to sensory conflict induced by virtual environment exhibits a better adaptation in older adults’ muscle response. Muscle response delay is shorter in first 10 trials compared to last 10 trials suggesting the possibility of postural balance improvement using sensory reweighting in older adults [90]. These results indicate that knowledge of the mechanism underlying sensory reweighting in older adults may become a stepping stone in understanding falls and in the development of prevention strategies.

## Future research directions

As reviewed above, diverse visual motion stimuli have been extensively used to study their influence on postural balance control. We suggest that a systematic examination of individual differences in processing visual motion signals will widen our perspective on the visual risk factors for falls. However, evaluating individual differences in visual motion perception is not a simple matter, because the ability of visual motion perception has diverse aspects. Thus, simple measurement of the detection threshold to motion stimulus cannot provide a proper index of individual differences in visual motion perception. An approach that takes into account diverse aspects of visual motion processing is required. Here, we propose three well-known phenomena related to motion perception and postural balance control, which could potentially indicate novel directions for research.

### Spatial suppression in motion perception

In the “Visual motion perception and balancing” section, we discussed the notion that the effect of ageing on the sensitivity to motion signals depends on the properties of the motion stimulus. The sensitivity to a small, low-contrast motion stimulus deteriorated with age, but the sensitivity to a large, high-contrast motion stimulus improves [42]. In other words, older adults are better at detecting the direction of a large, high-contrast motion stimulus than young adults. It was suggested that the relatively poor performance of young adults in the task was caused by strong centre-surround antagonism in visual motion perception. Centre-surround antagonism in visual motion perception refers to the fact that the firing rate of motion-sensitive neurons in the MT (middle temporal area in the brain) decreases when the surrounding area of the receptive field is stimulated [91]. Thus, the sensitivity to the motion stimulus declines as the stimulus size becomes larger [43, 92].

It is possible that the sensitivity to a small, low-contrast motion stimulus and the sensitivity to a large, high-contrast motion stimulus, which reflect the magnitude of spatial suppression, are related to the fall risks in older adults in a completely different manner. Further investigation of this theory will clarify our understanding of the relationship between visual motion perception and fall risk.

### Theories of multisensory integration

Balance control involves several sensory modalities, including visual motion perception. In order to use the multi-modal sensory signals efficiently, the system should be able to integrate visual, vestibular, and somatosensory signals properly. Therefore, understanding the principles by which our balance control system integrates multisensory information is critical to understand human balance control and fall risk. However, as far as we are aware, the ability to integrate multisensory signals has not been discussed in the context of fall risk factors.

There are two well-supported theories of multisensory cue integration that might contribute to the understanding of balance control and fall risk. The first theory states that humans integrate multi-sensory signals in a statistically optimal manner [93]. Statistical optimality of cue integration implies that the perceptual system depends more on sensory signals with low uncertainty than sensory signals with high uncertainty. For example, it is expected that the balance control system relies more on the visual stimuli when the stimulus is clearly presented (e.g. during the daytime) than when the visual stimulus is unclear (e.g. at night). It is largely unknown how the uncertainty of sensory signals affects the sensory integration in balance control.

The second theory is known as the causal inference model of multisensory cue integration [94]. According to the theory, temporal and spatial distances between multisensory signals determine the degree of perceptual integration. When sensory signals from different modalities are spatially and temporally distant, the perceptual system processes the signals independently, and when sensory signals from different modalities are spatially and temporally close, the perceptual system integrates the signals. By applying the two general theories of multisensory integration to understand human balance control, we could better understand why and how the balance control system reacts to diverse patterns of visual, vestibular, and somatosensory signals. Among visual inputs, visual motion signals are highly correlated with the vestibular and somatosensory signals. Thus, multisensory integration is particularly important in understanding how visual motion signals are related to balance control and fall risk.

Older adults who are prone to falls have a tendency to over-integrate multisensory signals [95, 96]. This is demonstrated in the sound-induced flash illusion, in which the number of concurrently presented sounds affects the perceived number of flashes. Subjects perceive two visual flashes when one visual flash and two consecutive auditory stimuli are briefly presented. In general, the delay between visual and auditory stimuli diminishes the effect. However, the older adults who are prone to falls report the illusion even when the delay between visual and auditory stimuli is relatively long. This result suggests that the risk of falls may be associated with the inability to infer the causes of multisensory signals [95].

Understanding the mechanism of multisensory integration in postural control is important in studying fall risk and developing prevention strategies. In a recent study, balance training intervention successfully improved postural balance control in healthy and fall-prone older adults, and the improvement was demonstrated to correlate with multisensory processing efficiency [97].

### Cognitive load and balance control

Many studies have examined the effect of cognitive load on postural control [98–103]. Cognitive spatial processing and postural balance control may rely on the same neural mechanics. When participants performed spatial and non-spatial memory tasks in sitting and standing positions in the Romberg test, a significantly higher number of errors were observed in the spatial memory task in the standing position compared to the sitting position. However, in the non-spatial memory task, the number of errors was not significantly different in the standing and sitting positions [104]. Postural sway was also affected by a secondary cognitive task [105]. Young and older adults without a history of falls exhibited significantly larger postural sway in a sentence completion task. However, older adults with a history of falls exhibited significantly larger postural sway in both the sentence completion and perceptual matching tasks. Moreover, the study also found that the surface condition (fixed vs swaying) had no significant effect in either age groups. The results suggested that the task of postural control shared cognitive resources with other cognitive tasks and that fall risk in older adults was related to a lack of cognitive resources. Similar results were also observed when cognitive task was demanding attention [106]. Participants were instructed to verbally classify an auditory tone as being high or low during several conditions of balance tasks: a combination of two ground conditions, sway and fixed, and three visual conditions, open, closed, and visual motion. In healthy older adults, postural balance control was affected when visual and somatosensory information was removed simultaneously. However, in balance-impaired older adults, the auditory task affected postural balance control in all sensory conditions. In contrast, Swan et al. [103] demonstrated that adding cognitive load decreased postural sway. In their study, subjects were required to stand still while performing cognitive tasks under different visual (open vs closed eye) and ground (fixed vs swaying pedal) conditions. Cognition tasks were spatial or non-spatial memory tasks. Surprisingly, the results indicated that in the closed-eyes/swayed-ground condition, which was the most difficult condition, cognitive tasks reduced the postural sway. This effect only appeared in the older adults group. Using the same memory task as one of the secondary tasks, Bergamin et al. [107] also observed improvement in postural stability in older adults by adding visuospatial tasks while maintaining balance. Recently, a cognitive task was also reported to decrease postural sway in young adults [108, 109]. Diverting attention from postural control decreases postural sway during the continuous cognitive task but not during the irregular cognitive task [108]. The continuous cognitive task in this study consisted of mentally counting and summing a series of three-digit numbers and mentally performing a series of simple mathematical equations. The irregular cognitive task consisted of verbally responding to random auditory stimuli and distinguishing high-pitched beeps from low-pitched beeps by verbally responding only to the high-pitched beeps [108]. In line with the findings of the studies mentioned above [103, 107–109], it has been shown that postural sway is smaller when subjects focus attention on a cognitive task compared to when subjects focus attention on postural control itself. In the study, participants were asked to silently count numbers in the cognitive task condition, and participants were asked to minimize movement of the hips in the postural control condition [110].

Although inconsistent findings regarding the effect of cognitive load on balance control necessitate further investigation, it seems clear that maintaining posture requires significant cognitive resources. We suggest that one of the main reasons for the sizable effect of cognitive load on balance control is that visual motion processing requires cognitive resources. The motion signal detected from a retinal image is an ambiguous signal. Retinal motion can represent movements of the head, eye, or objects in the world, and identifying the causes of retinal motion signals in sufficiently short duration would require sizable cognitive resources. Indeed, it has been demonstrated that the ability to detect briefly presented visual motion signals is a good indicator of IQ [111]. We suggest that further research on the relationship between cognitive load, visual motion perception, and fall risks may reveal one of the key factors responsible for the falls in older adults.

## Conclusion

Numerous studies have been conducted to identify relevant factors that contribute to the high risk of falls. It has been demonstrated that the deteriorations of diverse perceptual, cognitive, and muscular functions are correlated with fall risk. However, the majority of research regarding fall risk has focused on the simple correlation between the risk factors and the frequency of falls and did not attempt to provide clear mechanistic explanations regarding why and how those factors are related to falls. On the other hand, there are rich theory-oriented studies that examine how the balance control system is affected by sensory and cognitive processing. Researches on vection, sensory reweighting, spatial suppression in visual motion perception, multi-sensory integration, and effect of cognitive load on balance control are representative examples.

Considering that falls occur when one loses the control of balance, it is worth noting that the results of balance control studies have not been exploited in studies focusing on risk factors for falls. The lack of communication between the two fields is demonstrated by the fact that the ability of visual motion perception, which directly affects balance control, has not been studied as a key risk factor for falls. We believe that efforts to integrate knowledge from theory-oriented balance control studies and knowledge from application-oriented fall risk studies will be fruitful and help us to prevent falls more efficiently.

## Abbreviations

3D: Three dimensional
ETDRS: Early Treatment of Diabetic Retinopathy Study
GVS: Galvanic vestibular stimulation
IQ: Intelligence quotient
MAE: Motion after effects
MT: Middle temporal
MTF: Modular transfer function
WHO: World Health Organization
